# Supplementary material for: Intraspecific Genetic Variation for Behavioral Isolation Loci in Drosophila
Source: Genes (Basel). 2021 Oct 26;12(11):1703. doi: 10.3390/genes12111703 (PMC8619000; doi:10.3390/genes12111703)
Supplement: Supplementary file 1 [file genes-12-01703-s001.zip › genes-1412154-supplementary.pdf]

genes

MDPI

Supplementary Materials

Intraspecific Genetic Variation for Behavioral Isolation Loci in *Drosophila*

Jessica A. Pardy [1], Samia Lahib [1], Mohamed A. F. Noor [2] and Amanda J. Moehring [1,*]

[1] The University of Western Ontario, Department of Biology, London, ON, Canada; jpardy4@uwo.ca (J.A.P.), slahib@uwo.ca (S.L.).
[2] Duke University, Biology Department, Durham, NC, Canada; noor@duke.edu.
* Correspondence: amoehrin@uwo.ca

Table S1. Molecular markers used in QTL mapping the third chromosome.

Marker Name	Assay #1 or #2	Cyto. Location[1]	Primer Sequence[2]	*D. simulans* Line[3]
3L_173	2	61B	GGACATAATTTACGCAAGA, GACGGATTTGCCAAACAAAC	All
3L_1457	1, 2	62B	TGGAGAGCGGCGTTCCCCTG, TGGGCCACCTGTGGGCGTGGT	All
3L_3126	1,2	63E	GCCTAACCATTTCATTTGGT, GTTTCGAAAAGCCTCTCCTCACAC	197, 198, 199, 216
3L_3484	1,2	63E	GAGGACGGCGGTACATGAG, TAGTTCGTGGGCAGTAGCTC	167, 194, 196, FC
3L_10062	1	67E	TGGCAAACGAAACTGAAATC, GTTTACGATGGGAATGAAAATGGA	167, 196, 216, FC
3L_10365	1	67E	GACCCGAGAGCATTCTTGAG, GTTTTCCCTGCCCAAGAGACAATTA	194, 197, 198, 199
3L_16008	1,2	73C	CCAAGGGGCAGAAATAGGTA, CTTTGGAGCAACAATTGCATCAGA	All
3L_78D8	2	78D	TTTGAGTATCGCTTGGATGC, GCGGACCATTTAAATTCGAG	All
3R_668	1	82D	ATCAGCGCATTTCCCTACAC, TCGGCAAAATGTGATTTTCA	197
3R_697	1,2	82D	CTCTTTCCGCTCCCCTTA, GTTTGGAGATGCCAAACGAAATA	1: 216, FC; 2: All
3R_84D	1	84D	CACGACGTTGTAAAACGACAAAAAACTG-CATTTGGC, GAGAGCAGAAATCGAGAATCAGGC	FC
3R_3880	1,2	84E	CCTCCTTGGAATGATCCTCA, ATTATCCAAGTGCGGACGAC	1: 197, 216; 2: All
3R_8135	1	90B	GTTGCCAGCGGTAGTGTTTT, CCCTTTATGCCCACACATTC	All
3R_4051	2	93E	TTCTGTTATTGCCGCTGACA, ACTGCTTGCTCACCCAATCT	All except FC
3R_4012	2	93E	CGGGTTAATTGGACTTGCAT, GTTTCTGGCCAAGTCGAGAAAAA	FC
3R_17066	2	94A	GCGATTGTGTGCGAGTGTAT, GGGGGATTTTGTTTGTCATC	All
3R_20144	2	95F	GAACAAGCCGGCATACAGAT, AGGCACATTTGGATTGGATT	All
3R_22436	1	97D	ACAAACAGAGGAGCGCAGAT,	All

3R_23001	2	97F	CAGCGACTTGTCATCGCTAA TAGCTGCCATCGAGTGTGTC, GTTTTGCGGCTAATGAGAGG	All
3R_27488	1,2	100E	TGTCGGTGATGTTGAGTCTAT, GTTTGCGTCTGTTGAATTGTGTATC	All

[1] The cytological location is based on the banding pattern of polytene chromosomes in *D. melanogaster*, numbered from 61 to 100 (80 at the centromere) for the third chromosome. Each number is further subdivided A to F.

[2] Primers are listed as the Forward primer and then the Reverse primer, shown 5′ – 3′.

[3] The *D. simulans* lines for which this marker was used; different markers were used for different lines if the within-species polymorphism overlapped the between-species polymorphism.

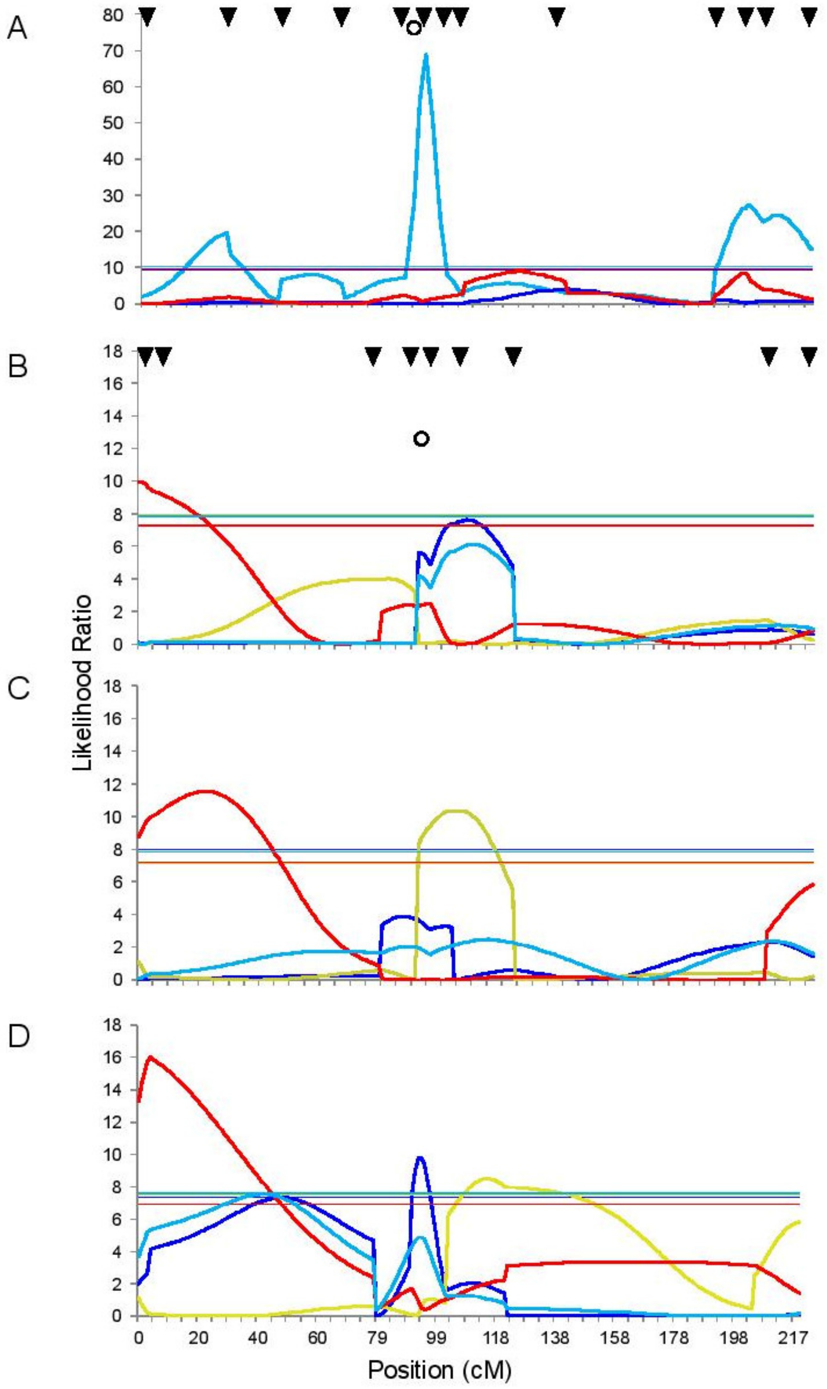

Figure S1. Likelihood ratios for *D. simulans* male traits that affect behavioral isolation with *D. mauritiana* females. The traits of courtship latency (yellow), copulation latency (dark blue), copulation occurrence (light blue) and copulation duration

(red) were scored in backcross (BC) male mapping populations of (**A**) BC$_{FC-ORIG}$ (adapted from Moehring *et al.*, 2004), (**B**) BC$_{FC}$, (**C**) BC$_{197}$ and (**D**) BC$_{216}$. Horizontal lines of the same color represent the significance thresholds for each trait in each population, as calculated by permutation analysis. Black triangles at the top of the figure represent the locations of the molecular markers used for genotyping; the cM (recombination) scale for the horizontal axis has less spread near the centromere and telomeres, making the markers appear overly clustered. The open circle represents the location of the centromere. Note that there is a different scale for the Y axis in panel (**A**) compared to the other panels.

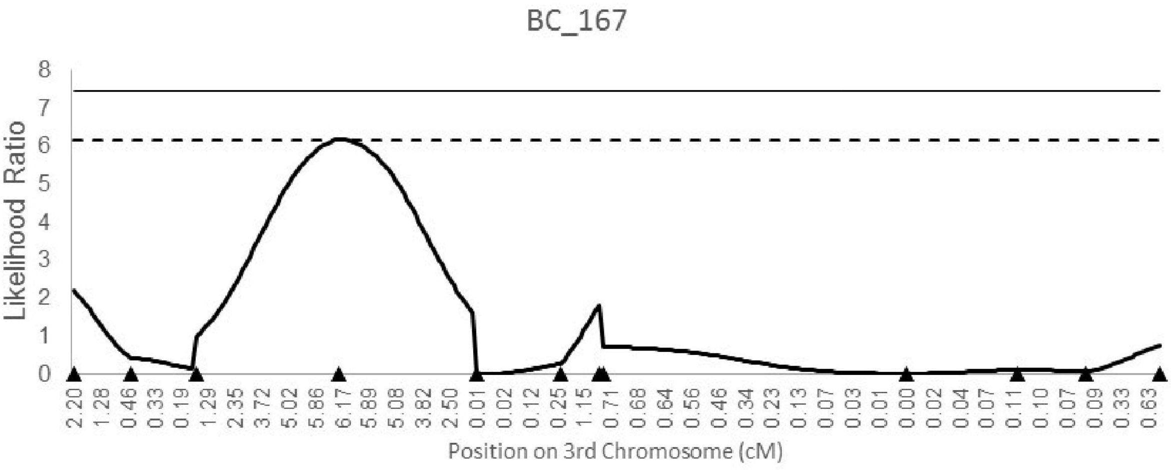

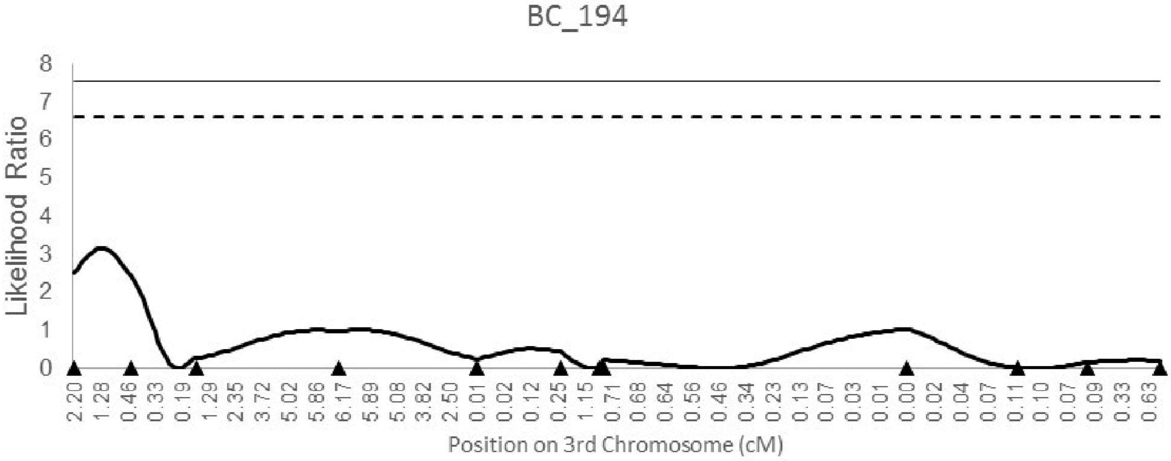

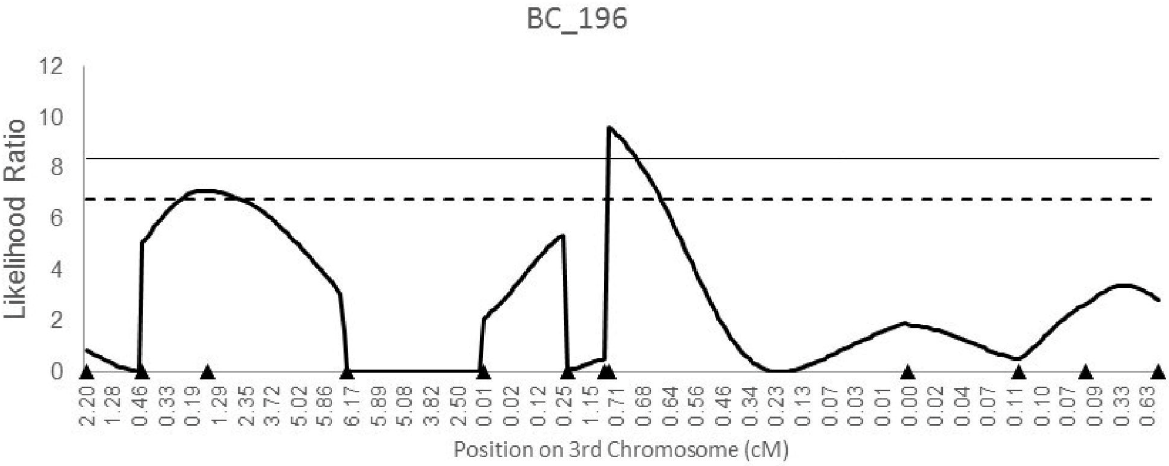

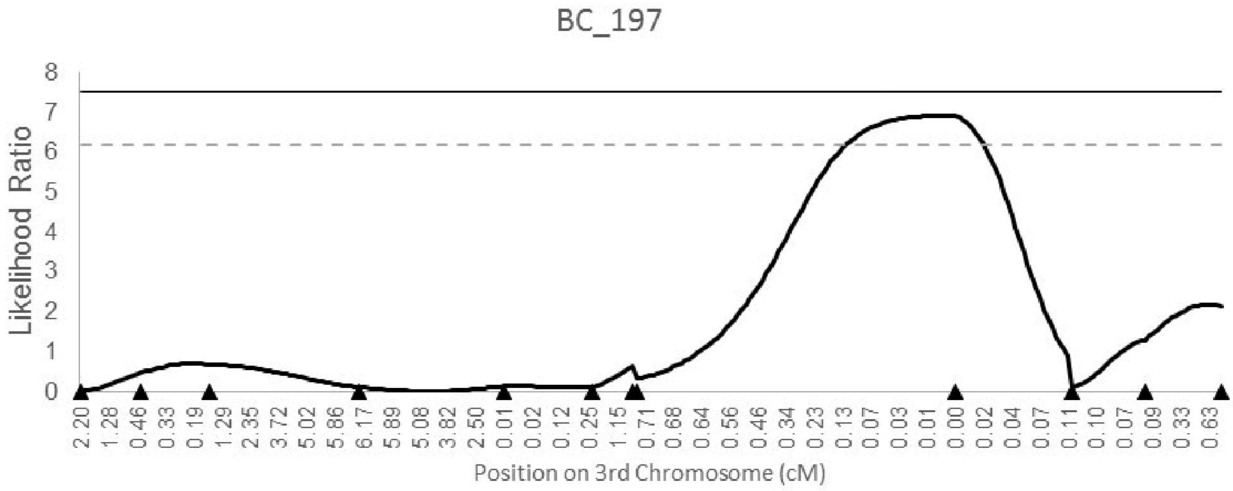
BC_197
Likelihood Ratio
0 1 2 3 4 5 6 7 8
2.20 1.28 0.46 0.33 0.19 1.29 2.35 3.72 5.02 5.86 6.17 5.89 5.08 3.82 2.50 0.01 0.02 0.12 0.25 1.15 0.71 0.68 0.64 0.56 0.46 0.34 0.23 0.13 0.07 0.03 0.01 0.00 0.02 0.04 0.07 0.11 0.10 0.07 0.09 0.33 0.63
Position on 3rd Chromosome (cM)

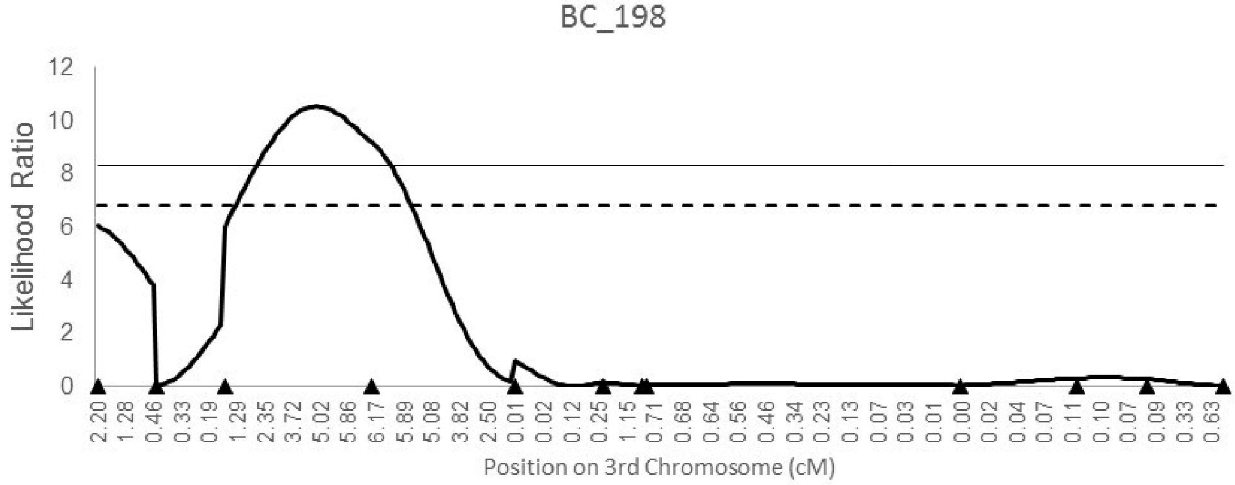
BC_198
Likelihood Ratio
0 2 4 6 8 10 12
2.20 1.28 0.46 0.33 0.19 1.29 2.35 3.72 5.02 5.86 6.17 5.89 5.08 3.82 2.50 0.01 0.02 0.12 0.25 1.15 0.71 0.68 0.64 0.56 0.46 0.34 0.23 0.13 0.07 0.03 0.01 0.00 0.02 0.04 0.07 0.11 0.10 0.07 0.09 0.33 0.63
Position on 3rd Chromosome (cM)

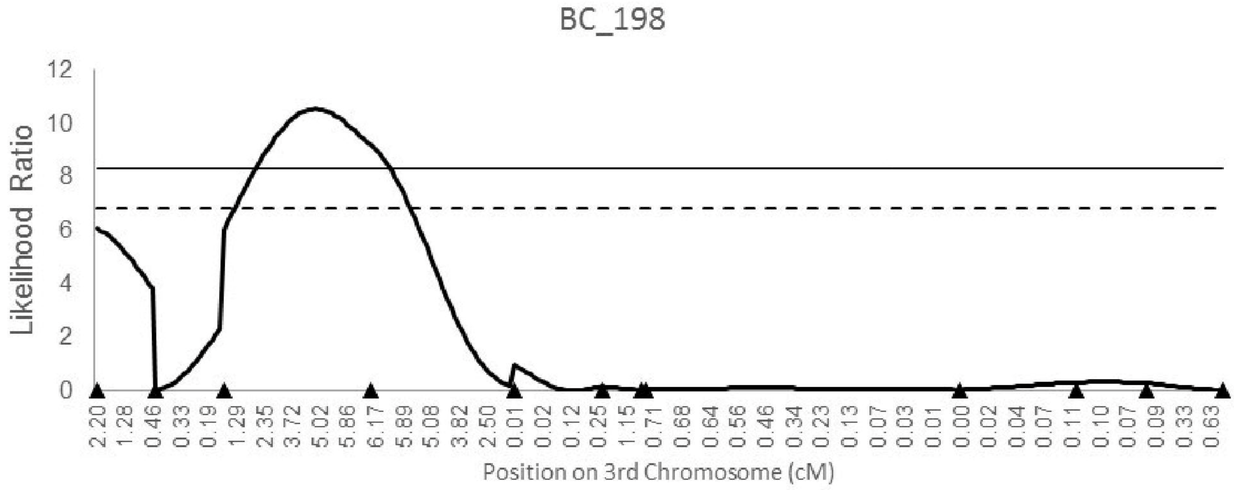
BC_198
Likelihood Ratio
0 2 4 6 8 10 12
2.20 1.28 0.46 0.33 0.19 1.29 2.35 3.72 5.02 5.86 6.17 5.89 5.08 3.82 2.50 0.01 0.02 0.12 0.25 1.15 0.71 0.68 0.64 0.56 0.46 0.34 0.23 0.13 0.07 0.03 0.01 0.00 0.02 0.04 0.07 0.11 0.10 0.07 0.09 0.33 0.63
Position on 3rd Chromosome (cM)

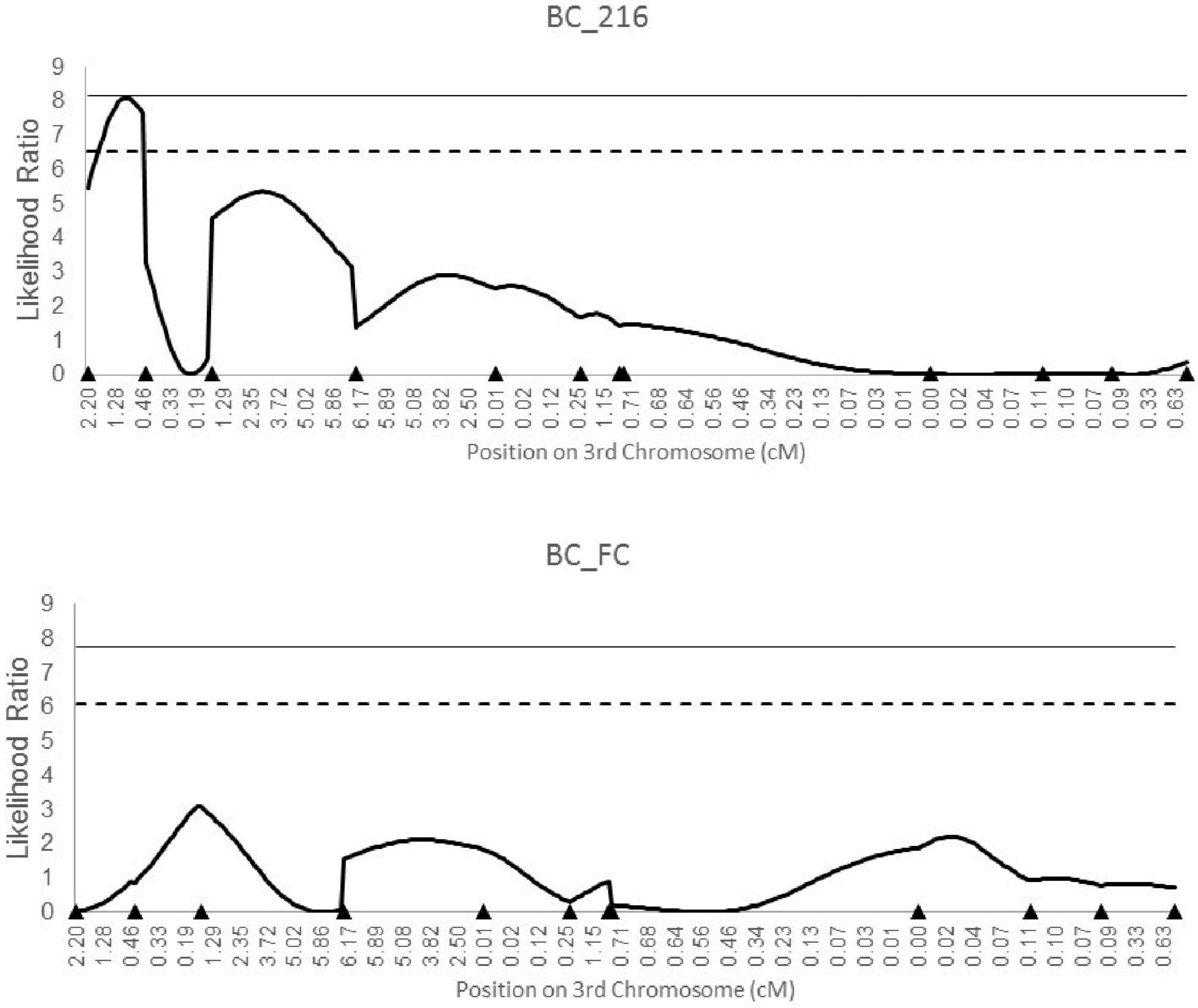

Figure S2. Likelihood ratios for the male trait of copulation occurrence for BC males from eight strains of D. simulans when paired with D. mauritiana females. Triangles along the x-axis represent molecular marker locations. The 0.05 threshold is shown as a solid horizontal line; the 0.10 threshold is shown as a dashed horizontal line.
